# Transformations of PTCDA structures on rutile TiO_2_ induced by thermal annealing and intermolecular forces

**DOI:** 10.3762/bjnano.6.155

**Published:** 2015-07-10

**Authors:** Szymon Godlewski, Jakub S Prauzner-Bechcicki, Thilo Glatzel, Ernst Meyer, Marek Szymoński

**Affiliations:** 1 Faculty of Physics, Astronomy and Applied Computer Science, Jagiellonian University, Łojasiewicza 11, 30-348 Krakow, Poland; 2 Department of Physics, University of Basel, Klingelbergstr. 82, 4056 Basel, Switzerland

**Keywords:** PTCDA, TiO_2_, rutile, self-assembly, STM

## Abstract

Transformations of molecular structures formed by perylene-3,4,9,10-tetracarboxylic dianhydride (PTCDA) molecules on a rutile TiO_2_(110) surface are studied with low-temperature scanning tunnelling microscopy. We demonstrate that metastable molecular assemblies transform into differently ordered structures either due to additional energy provided by thermal annealing or when the influence of intermolecular forces is increased by the enlarged amount of deposited molecules. Proper adjustment of molecular coverage and substrate temperature during deposition allows for fabrication of desired assemblies. Differences between PTCDA/TiO_2_(110) and PTCDA/TiO_2_(011) systems obtained through identical experimental procedures are discussed.

## Introduction

Molecular self-assembly appears to be a very powerful and versatile tool for the formation of molecular structures and devices at the nanoscale. Thus, it continuously attracts much attention in the field of nanoscience and nanotechnology [1–7]. An insight into the fundamental physical and chemical mechanisms leading to the formation of self-assembled architectures allows for proposing a means of control over that spontaneous process or for suggesting further post-processing methodology in the search of desired material.

Among many different substrates that are used as templates for molecular growth, metal oxides are currently regarded as very promising supports. For example, titanium dioxide surfaces are exceptionally useful in various applications, including the catalysis, solar energy conversion, gas sensing and others [8–15]. Merging two classes of materials, i.e., metal oxide surfaces with organic molecules, seems to be one of the most challenging and encouraging directions in the surface science of oxide materials [14–18].

Studying prototypical systems gives us a chance to recognize the basic elements of the investigated phenomena and to identify underlying interactions. The necessity of understanding model systems becomes even more evident when combined materials, such as organic molecules adsorbed on a metal oxide surface, are examined. Thus, we have decided to perform our research on such a model system, comprising perylene-3,4,9,10-tetracarboxylic dianhydride (PTCDA) molecules adsorbed onto the (110) face of rutile titanium dioxide. The chosen perylene derivative, i.e., PTCDA, is often considered as a model planar-stacking organic molecule for organic semiconductors [19–26], similar to how the CO molecule is regarded as a model for small inorganic molecules. There are several important factors giving rise to such recognition of PTCDA molecules. First, PTCDA belongs to the group of intensive dyes and is applied in a wide range of optoelectronic devices, such as photosensors and organic light emitting diodes. Second, as flat molecules with delocalized π orbitals and a permanent quadrupole moment, the molecules are likely to adsorb flat on crystalline templates and form ordered structures through self-assembly processes. Third, the PTCDA molecules are easily processable. They exhibit high thermal stability, as well as robustness against electron and photon bombardment. Therefore, one may analyse structures composed of PTCDA molecules with various experimental techniques [19–20,27]. With respect to the substrate of interest, the (110) face of the rutile titania is the most often studied surface of TiO_2_ [14–16]. Thus, it is quite often recognized as the exemplary transition metal oxide surface.

Needless to say, numerous studies on PTCDA molecules address their adsorption and layer formation on various substrates. Usually, when molecule–molecule interactions dominate the layer development, the molecules are ordered in a herringbone structure closely resembling PTCDA bulk crystal layers [20,28]. Such an arrangement was reported, for example, on Ag(111) [19]. In the case of increased molecule–substrate interactions, brick-wall structures also formed, such as, for instance, on Ag(110) [19,29]. However, on highly reactive substrates exhibiting a large density of dangling bonds exposed at the vacuum interface, such as InAs, Si or Ge, strong molecule–substrate coupling hinders the growth of any regular structure. Interestingly, the introduction of a single atomic layer of hydrogen dramatically changes the situation in favour of molecule–molecule interactions and allows for the formation of nanocrystalline islands [30–34].

To the best of our knowledge, to this day only a few studies contribute to the characterization of the PTCDA/TiO_2_ system. Namely, Komolov et al. [35] analysed few-nanometre thick PTCDA layers on the TiO_2_(110) surface by employing spectroscopic techniques and therefore did not provide any information on sub- and monolayer ordering. The influence of dispersion forces on supramolecular ordering was described by Godlewski et al. [36]. The properties of the multilayer of PTCDA molecules on the (110) face were addressed by Cao et al. [37]. Later, the same authors discussed the charge transfer timescale at the PTCDA/TiO_2_(110) interface with respect to the molecular orientation [38]. Tekiel et al. [39] presented scanning tunnelling microscopy (STM) studies of the PTCDA/TiO_2_(011) system focused on molecular chain formation. The molecules were arranged in chains when deposited at sub-monolayer coverage on a surface kept at specific temperatures from a very narrow window of substrate temperatures. However, neither the nature of chain formation nor the mechanism of molecule–substrate binding has been explained so far. Higher densities of molecular material on the TiO_2_(011) surface have also been studied [17]. Unfortunately, the first layer is only partially ordered, and many disordered areas have been found in it. The formation of the second well-ordered layer was considered questionable, making the PTCDA/TiO_2_(011) system not very promising from the point of view of optoelectronic applications [17].

In the following, we extend previous research on the PTCDA/TiO_2_(110)-(1 × 1) system [36]. Godlewski et al. [36] essentially paid attention to the influence of dispersion forces (changed by the density of molecules on the surface) on the formation of the ordered layer. In contrast, our main concern here is the transformation of the molecular structure driven by thermal annealing. Thus, we systematically analyse the impact of post-deposition annealing and deposition at elevated temperatures on the self-assembly processes for different adsorbate coverage values. The formation of molecular meandering lines at moderate coverage and the creation of ordered brick-wall-like structures of the closed layer are presented. The role of post-deposition annealing in triggering transformations between the obtained structures is discussed. In conclusion, we compare experiments on adsorption of PTCDA molecules on the two stable rutile titania surfaces, i.e., the (110) and (011) faces, pointing to differences in molecular behaviour with respect to these two substrates.

## Results and Discussion

### The TiO_2_(110)-(1 × 1) surface

**Figure 1:**
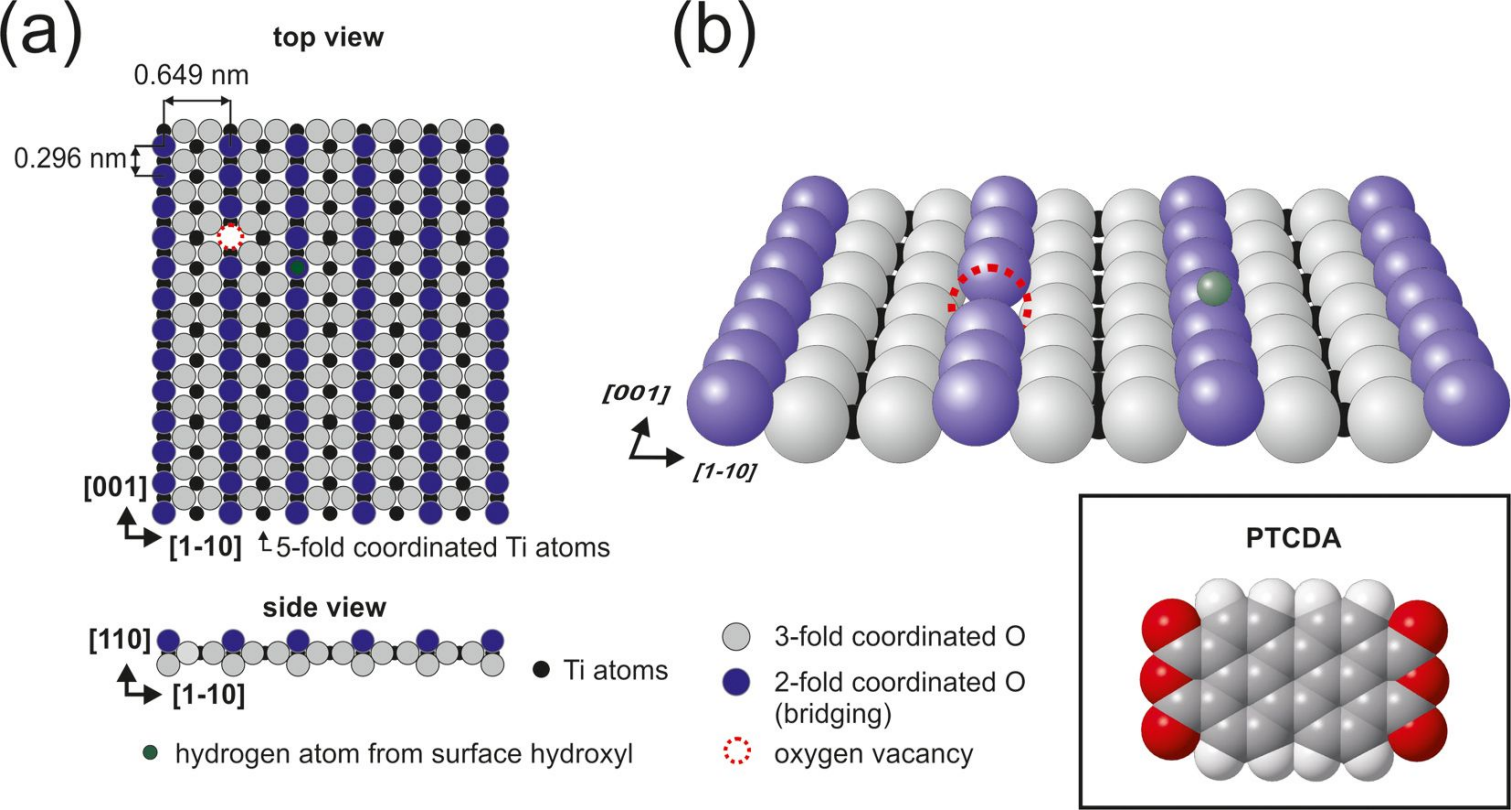
Structural model of the TiO_2_(110)-(1 × 1) surface: (a) top and side view, (b) perspective view. The inset in panel (b) shows the structural model of a PTCDA molecule (colour coding: grey – carbon, red – oxygen, white – hydrogen).

The TiO_2_(110)-(1 × 1) surface model has been well established on the basis of experimental and theoretical studies [14]. The (110) face is the most stable face of rutile titanium dioxide. The surface is composed of protruding oxygen rows running along the [001] crystallographic direction separated by approximately 0.649 nm. The structural model of the surface is shown in Figure 1. Despite the presence of outstanding oxygen atoms, the STM mapping is largely dominated by electronic effects. As a result, the bright rows running along the [001] direction that are clearly visible in the STM images correspond to titanium rows, while the oxygen rows are located in between. The overall STM appearance is much different from the surface topography. Additional bright spots recorded within dark oxygen rows are attributed to oxygen vacancies (fainter spots) or single and double surface hydroxy groups (brighter spots). The oxygen vacancies are created during the preparation procedure, leading to a slight reduction of the sample and decreasing the intrinsic band gap from approximately 3.0 eV to 1.5–2.5 eV. Hydroxy groups are created as a result of atomic hydrogen adsorption and dissociative adsorption of water at oxygen vacancies. The surface is very sensitive to the presence of water among residual gases and therefore the maintenance of the high vacuum level during sample preparation plays a crucial role. The adopted experimental procedure enables us to obtain clean surfaces with several oxygen vacancies and some hydroxy groups after final annealing. However, the subsequent deposition of organic molecules requires keeping the sample in the vicinity of the heated effusion cell. This results in enhanced water adsorption on the surface, leading to passivation of oxygen vacancies and formation of a great number of surface hydroxy groups. Therefore, covering the surface with hydroxy groups is the inevitable drawback of the evaporation processes. For the sake of completeness, a scheme of the PTCDA molecule is shown in the inset of Figure 1b.

### Single molecules

**Figure 2:**
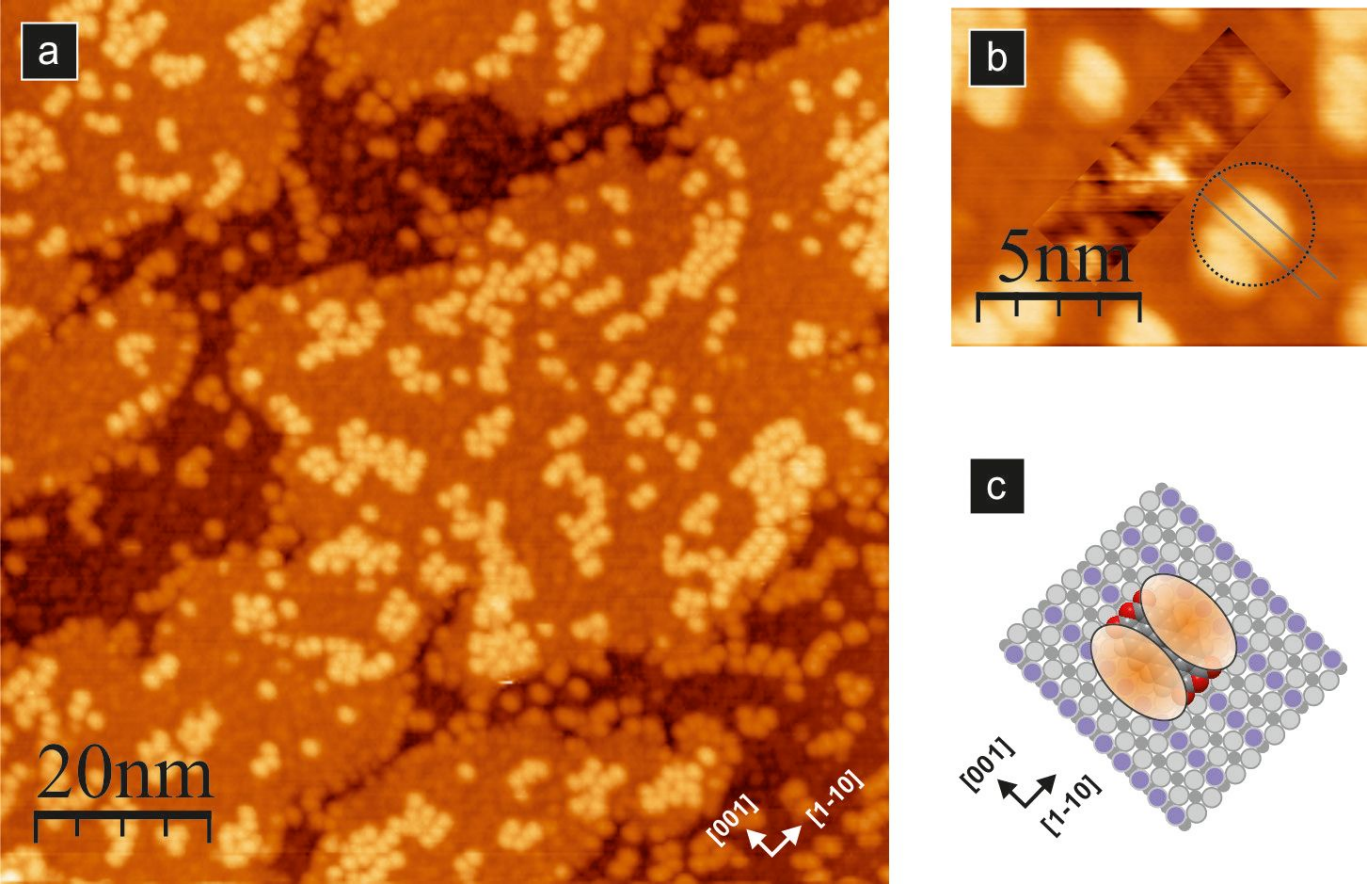
Empty-state STM image of a low coverage (<0.05 ML) of PTCDA molecules assembled on the TiO_2_(110) surface (a); (b) magnified image showing the position of the PTCDA molecule relative to the surface rows; for clarity, the image contrast inside the rectangle is adjusted to improve the visibility of surface reconstruction rows, and a single molecule STM image (two lobes) is marked with a dashed circle. (c) The structural model of the PTCDA molecule on the surface, where two ellipses mimic the STM appearance of a single molecule; all images acquired with 2 pA tunnelling current and 2.0 V bias voltage.

A typical STM image of the low coverage of PTCDA molecules assembled at room temperature on TiO_2_(110)-(1 × 1) is shown in Figure 2. We define a monolayer coverage (1 ML) as the coverage of the closed layer with chemisorbed molecules, and then the density of molecules reaches 1 molecule per 117 Å^2^ [36]. Accordingly, in Figure 2, the molecular coverage is less than 0.05 ML. As is clearly visible, the substrate surface after molecule deposition contains a considerable number of hydroxy groups. In Figure 2, these groups are seen as small faint spots, whereas PTCDA molecules are imaged as larger and brighter oval features. The fact that nearly all surface steps are entirely covered with PTCDA molecules indicates high mobility at room temperature that enables diffusion towards terrace edges. There is, however, a substantial number of molecules trapped at terraces. They do not form any ordered structures and are likely pinned by surface defects. The latter observation is further supported by the high stability of the system against scanning with different bias voltage and tunnelling current parameters, which do not trigger any type of tip-induced manipulation process. Despite the position, i.e., on a step-edge or on a terrace, all molecules are oriented in the same way, i.e., along the [001] crystallographic direction. This indicates that in adopting the most favoured geometry upon adsorption, the key role is played by the molecule–substrate interactions. For on-terrace species, immobilization is reached by additional interactions with surface defects.

For low, sub-monolayer coverage, neither the deposition of molecules at elevated temperatures nor the post-deposition annealing have any significant influence on the system. Generally, the molecules appear as single bright lobes and no intramolecular features are visible (Figure 2a). The lobes are located centrally on top of the outstanding oxygen rows. However, when a higher resolution is achieved the single molecule is imaged as a characteristic double lobe feature, as noted by a dashed circle in Figure 2b. This finding enables us to determine the molecule orientation on the surface, as the lowest unoccupied molecular orbital (LUMO) should contribute mostly to the STM appearance when imaging of empty states is performed. The charge density contour of the PTCDA LUMO orbital comprises 14 lobes located symmetrically with respect to the longer molecular axis, where the orbital wave function changes its sign. As a result, the charge density in the proximity of the longer axis is very low, and thus, that area does not contribute significantly to the tunnelling process. In our high-resolution measurements, a single PTCDA molecule appears as two lobes with a clear depression between them. Considering the LUMO contour, the depression could only be assigned to the longer axis of the molecule, similar to previous studies on Ag/Si(111) [40] and TiO_2_(011)-(2 × 1) [39]. Therefore, we conclude that the longer molecular axis is oriented parallel to the reconstruction rows of the substrate. The proposed geometry has also been supported previously by theoretical calculations [36], which showed preferential orientation of the longer molecule axis along the surface rows and a very small barrier for movement especially in the [001] crystallographic direction.

### Higher coverages at room temperature

**Figure 3:**
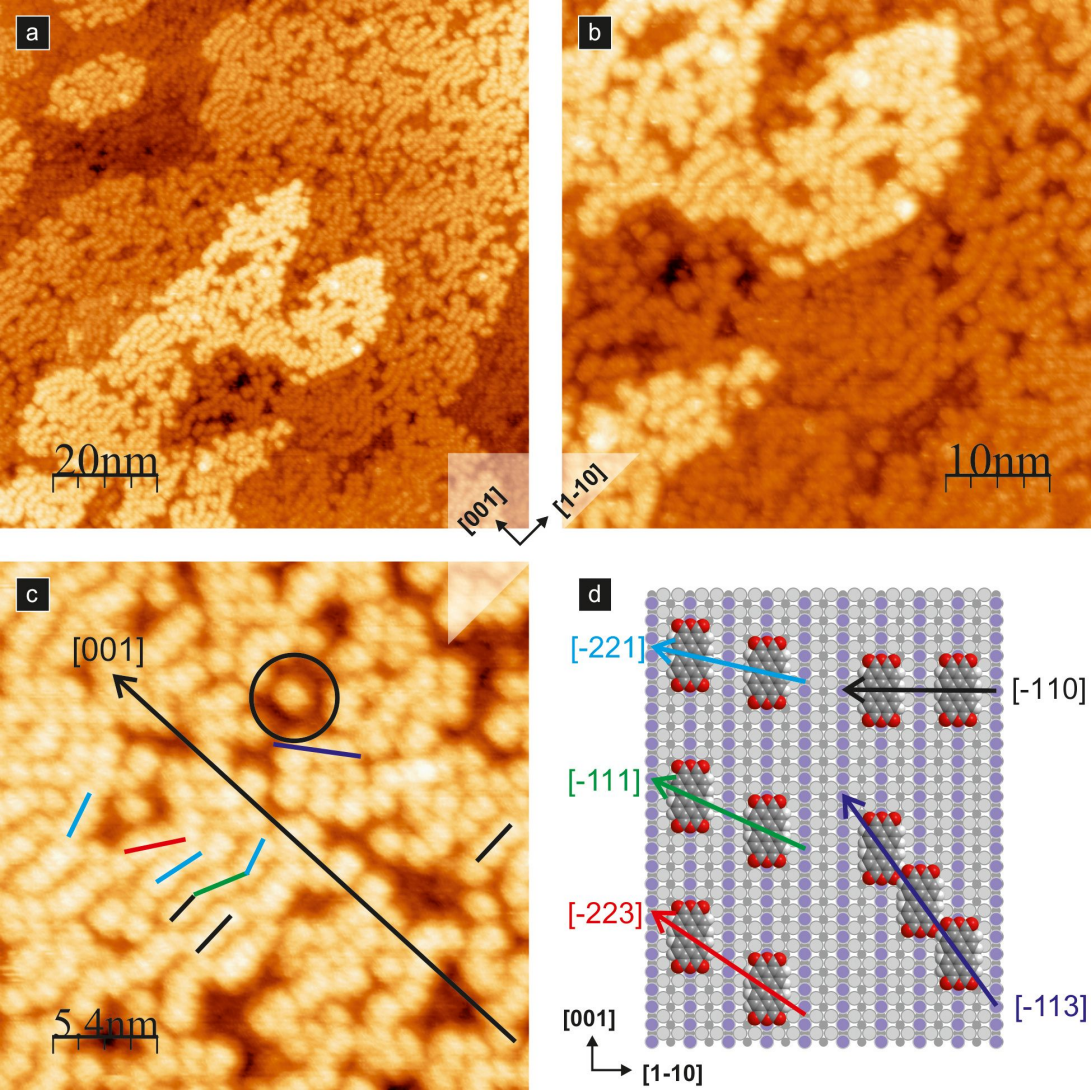
0.7 ML of PTCDA molecules on a TiO_2_(110) surface; (a), (b) and (c) are empty state STM images showing meandering molecular lines, and (d) shows the structural model of molecular lines. Coloured arrows (panel (d)) and lines (panel (c)) indicate different orientations of neighbouring molecules, and a circle indicates a single molecule STM image containing two lobes.

When more molecules are deposited, the intermolecular binding gives rise to the formation of supramolecular structures. The first example is presented in Figure 3, where meandering molecular lines are shown. They are created upon adsorption of a moderate number of molecules (in this case approximately 0.7 ML) at room temperature. It is worth noting that individual molecules are still oriented along the [001] crystallographic direction.

The observed meandering lines run across the surface rows. They comprise molecules located over every second titanium surface row. The positions of adjacent molecules are often shifted along the [001] crystallographic direction, i.e., surface rows, by a multiple of a surface unit cell dimension. The shifting distance seems to be accidental, and no correlation between the shifts of neighbouring molecular pairs is observed. Hence, the lines have a meandering distorted appearance. Nevertheless, along such a meandering line, one may distinguish pairs, triples and (rarely) larger numbers of molecules that locally form a straight line, adapting to a preferred crystallographic direction (see the black–green–light blue broken line in Figure 3c). If we assume that shifts between neighbouring molecules are possible by an integer multiple of the surface unit cell length (i.e., 0.296 nm), then we obtain different observed locations of PTCDA pairs. In Figure 3c and Figure 3d, the examples showing pairs of molecules shifted by one, two and three unit cell lengths, as well as non-shifted molecules, are indicated by light blue, green, red, and black lines and arrows, respectively. With the dark blue line and the arrow in Figure 3c and Figure 3d, we mark also the arrangement composed of molecules shifted by three surface lattice vectors but sitting on neighbouring rows. The formation of shifted molecular pairs is the result of relatively weak permanent quadrupole moment interactions and dispersion interactions [36]. Therefore, the meandering chains are not stable against thermal annealing, as will be discussed in the following sections. A detailed description of the intermolecular forces of PTCDA molecules adsorbed on the TiO_2_(110)-(1 × 1) surface may be found in [36].

**Figure 4:**
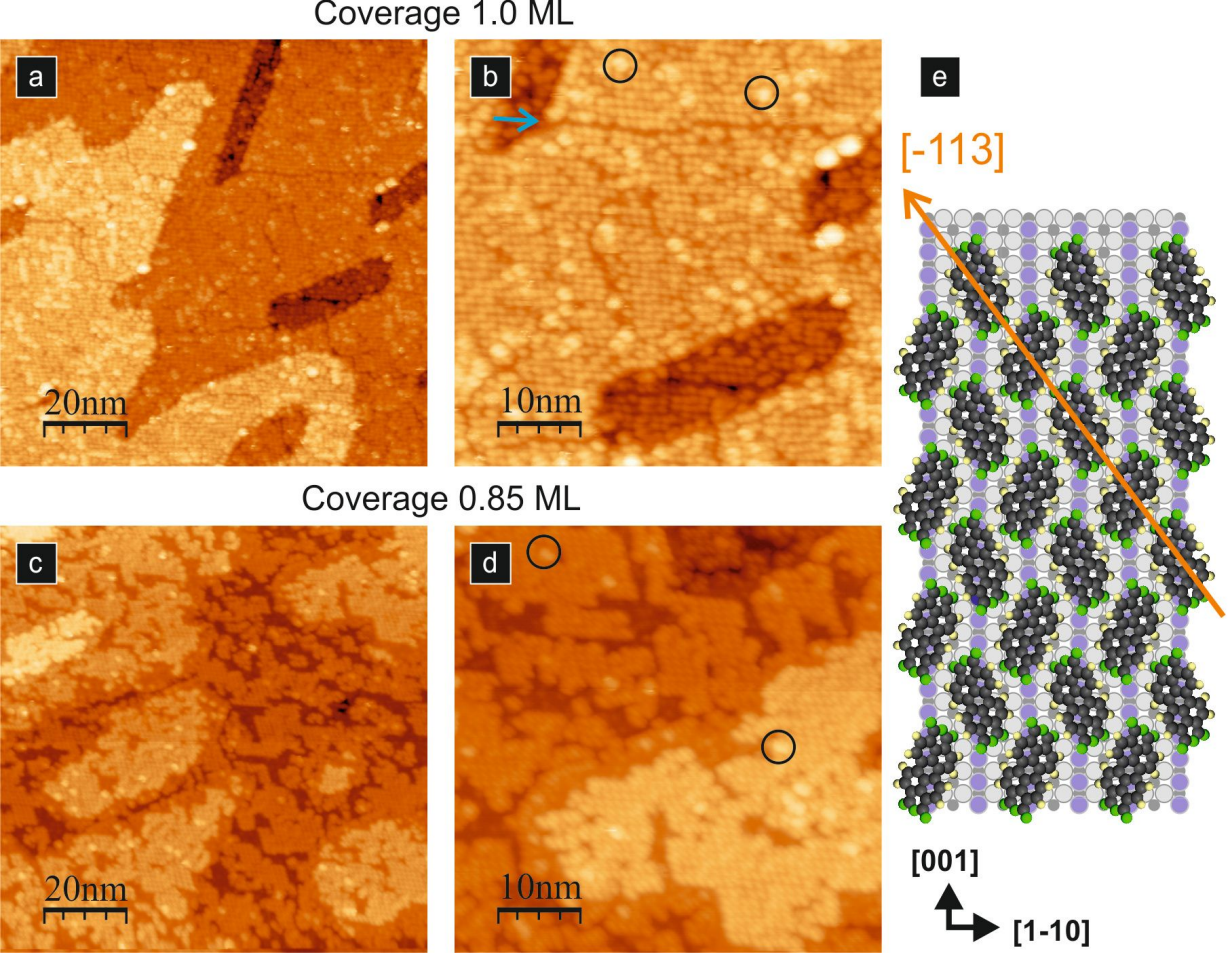
A well-ordered PTCDA layer formed at room temperature. (a) and (b): empty-state STM images of a closed layer, where the blue arrow indicates the domain boundary and black circles indicate molecules trapped on top of the first layer; (c) and (d): empty-state STM image of 0.85 ML coverage of PTCDA molecules, where black circles indicate molecules trapped on top of the first layer. All STM images acquired with 2.0 V bias voltage and 2 pA tunnelling current. (e) A structural model of an approx. 1.0 ML well-ordered PTCDA layer.

In Figure 4a,b and Figure 4c,d, we show the STM images of a closed layer and of 0.85 ML of PTCDA molecules adsorbed at room temperature, respectively. An increase in the amount of deposited molecular material leads to a transition in the behaviour of adsorbed molecules. At a coverage of 0.85 ML, we observe the formation of structures exhibiting the brick-wall-like pattern. The STM pattern is preserved also for a closed layer. As discussed by Godlewski et al. [36], the actual arrangement of molecules in the layer is a herringbone structure (see Figure 4e). The strikingly different STM appearance of the layers stems from the fact that upon increasing the amount of molecular material, the dispersion interactions emerge as decisive and the molecules within the layer become chemisorbed [36]. Such chemisorbed molecules are bent along the diagonal axis. In turn, the bending changes the STM appearance of each individual molecule from two lobes into a single straight lobe, and consequently, the layer exhibits a brick-wall-like pattern.

In principle, the STM images of the molecular layer exhibit the existence of the domain boundaries spreading out at distances reaching up to tens of nanometres. The exemplary domain boundary is noted by the blue arrow in Figure 4b. The additional bright dots marked with black circles on top of the layer (Figure 4, panels b and d) are attributed to single PTCDA molecules from the second layer. They are often located at the domain boundaries. The observed rearrangement of molecules from meandering molecular lines into a well-ordered layer upon increasing the coverage indicates a rather low stability of the meandering lines. The intermolecular dispersion forces are responsible for the formation of energetically favoured ordered structure [36].

### Post-deposition annealing

**Figure 5:**
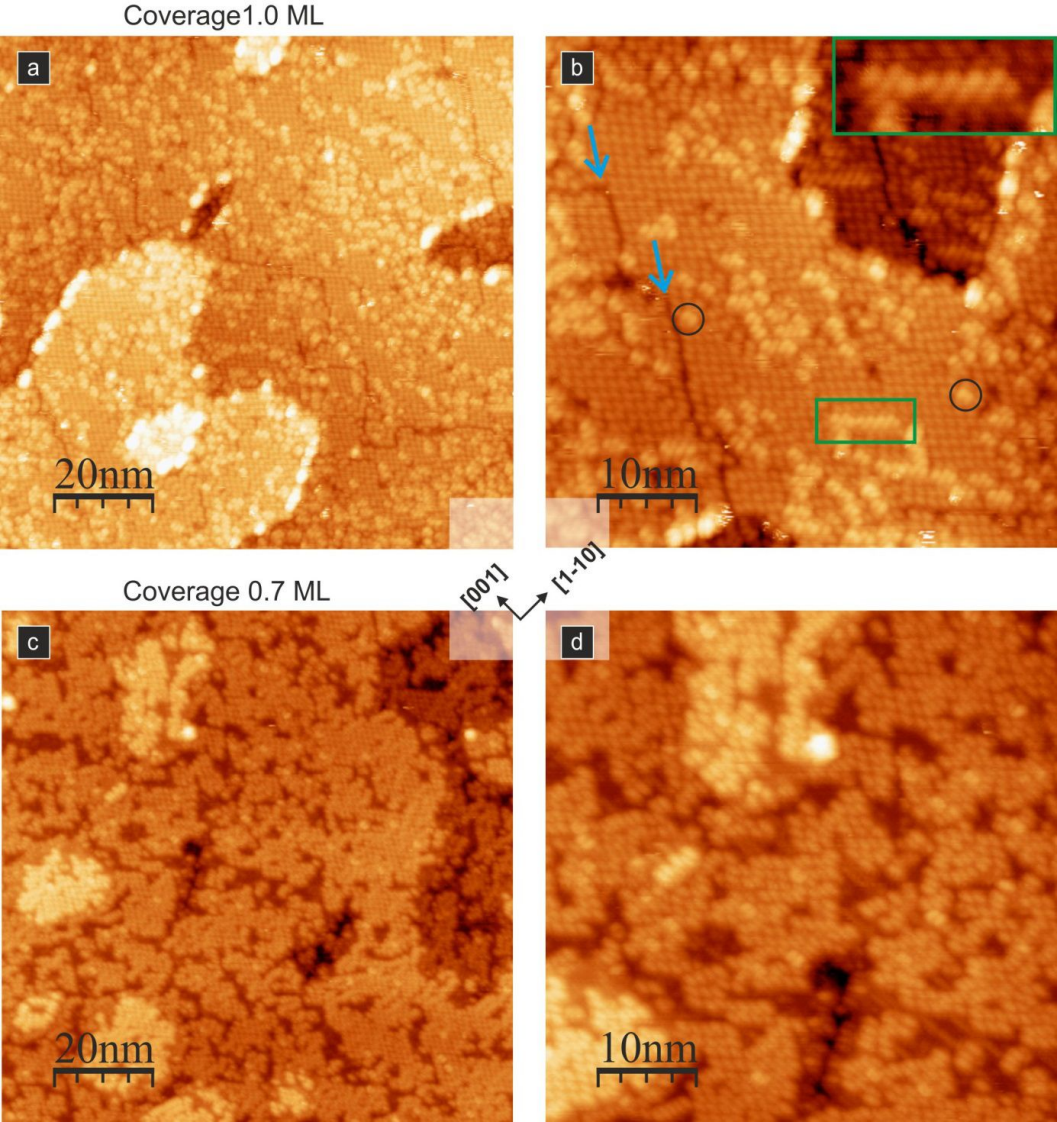
Empty-state STM images of the PTCDA molecules on the TiO_2_(110) surface after 100 °C annealing. (a) and (b): closed molecular layer (blue arrows mark domain boundaries, while black circles and a green rectangle mark admolecules on the top of the first layer); (c) and (d): 0.7 ML of PTCDA molecules. Well-ordered structures were obtained by the transformation of the meandering molecular lines shown in Figure 3 by thermal annealing; all images acquired with 2 pA tunnelling current and 2.0 V bias voltage.

To test the thermal stability of the system, we have performed post-deposition annealing experiments. The annealing of the closed molecular layer performed at 100 °C does not lead to any significant change in the molecular assembly. The results are shown in Figure 5a and Figure 5b. Due to a small excess of the monolayer coverage, additional admolecules located mainly at the defects of the first monolayer are visible. They exhibit a typical two-lobe appearance (see black circle in Figure 5b) and are oriented perpendicular to the PTCDA molecules within the closed layer below, i.e., the admolecules are placed with their longer axis pointing towards the [1−10] direction of the substrate. Additionally, short molecular lines of the second-layer molecules are formed due to intermolecular electrostatic and dispersion interactions, as indicated by the green rectangle and the inset in Figure 5b.

Interestingly, annealing the sample with the meandering molecular lines leads to a change in the ordering of the adsorbed molecules. We have annealed the sample that was presented in Figure 3 (before annealing) at 100 °C. The resulting structure is shown in Figure 5c and Figure 5d. The comparison of the STM images in Figure 3 and Figure 5c,d clearly illustrates the transformation of the meandering line structure with relatively weakly interacting molecules into a complex structure identical to the one for a closed layer. The observation proves that thermal annealing at 100 °C provides enough energy to overcome the energy barrier associated with the transformation into an ordered structure.

**Figure 6:**
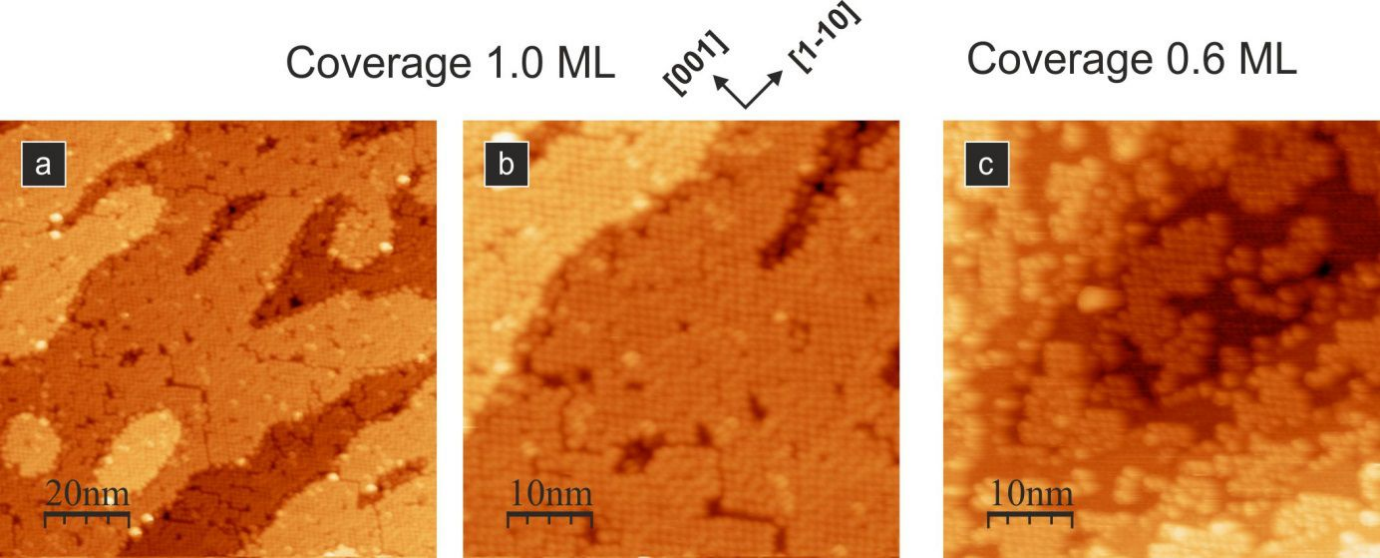
Empty-state STM images of the densely packed molecular structures. (a) and (b): closed layer PTCDA structure obtained when the molecules are adsorbed on the sample kept at elevated to 100 °C temperature; (c) 0.6 ML of PTCDA molecules adsorbed on the sample kept at 100 °C. All STM images acquired with 2 pA tunnelling current and 2.0 V bias voltage.

Formation of the densely packed regular structure is also observed when the molecules are adsorbed on the sample kept at elevated temperature. Figure 6 demonstrates the well-ordered molecular layer obtained when the substrate temperature during deposition was maintained at 100 °C. We see that the ordered layer is formed for a closed layer (Figure 6a,b) as well as for sub-monolayer coverage (Figure 6c, coverage of approx. 0.6 ML).

**Figure 7:**
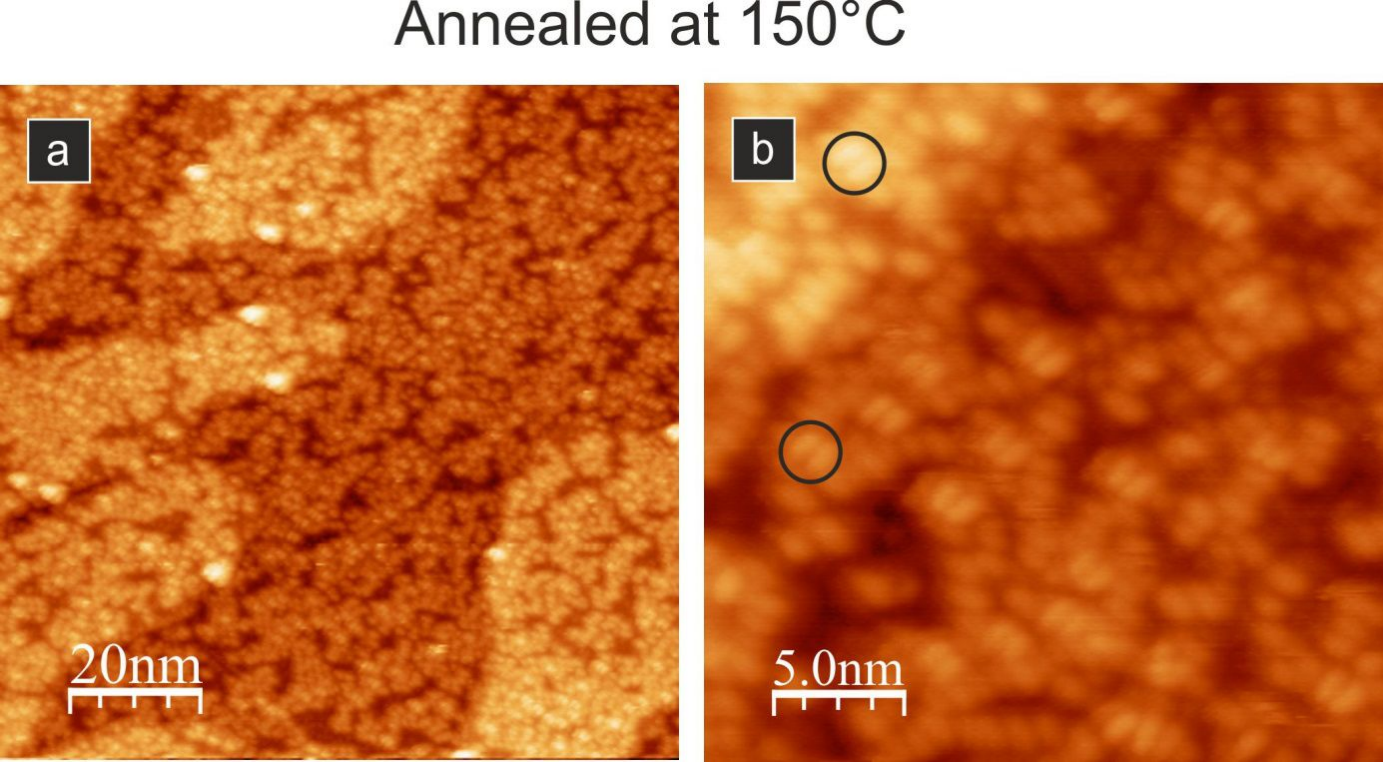
Empty-state STM images of the closed layer of PTCDA molecules after annealing to 150 °C: (a) 100 nm × 100 nm scan and (b) 25 nm × 25 nm scan. The layer is completely disordered, and the black circles in (b) indicate single molecules exhibiting typical two-lobe contrast. All images acquired with 2 pA tunnelling current and 2.0 V bias voltage.

Further experiments showed that the obtained ordered structure exhibiting a brick-wall-like pattern is also unstable if the sample is annealed at a slightly higher temperature. In Figure 7a,b, the closed molecular layer annealed at up to 150 °C is shown. We see that the previously observed structure is destroyed during annealing and the new layer is completely disordered, with clearly visible single molecules exhibiting typical two-lobe contrast (see black circles in Figure 7b). The molecules showing the two-lobe STM appearance are most likely located on top of other molecules. It seems that the observed layer is made of disarranged three-dimensional molecular clusters. We do not expect desorption of molecules at 150 °C; therefore, the initial 1 ML coverage should be maintained after annealing.

## Conclusion

We have performed STM measurements of molecular structures formed by PTCDA molecules on the surface of rutile TiO_2_(110). The experiments demonstrated that the molecular structures could be changed by thermal annealing of the system and also by the amount of molecules. At low coverage, the molecules adsorb mainly as single entities at surface steps and surface defects at terraces. At moderate coverage, the molecules deposited at room temperature form meandering molecular lines, which are transformed into the ordered molecular layer either by mild thermal annealing (approx. 100 °C) or by increasing the amount of molecules (>0.85 ML). The ordered layer is destroyed by annealing at higher temperatures (ca. 150 °C) resulting in a completely disordered phase.

In the analysed case, the self-assembly process is steered by thermal annealing, which provides additional energy, enabling equilibration. The method allows for switching from the formation of meandering, not well-organized chains to a well-ordered phase. Deposition of the molecules on a substrate maintained at an elevated temperature leads to the formation of similar structures. The likeness of these results indicates that the obtained structures are formed due to the equilibration of the system and not through kinetic trapping. At this point, it is worth comparing the behaviour of the molecules on the (110) surface with the other stable face of rutile titania, namely, the (011) surface. On the latter surface, post-deposition annealing does not lead at all to the formation of any ordered structures. The ordering is observed only if the molecules are deposited at the surface kept at a temperature of approximately 100 °C. Moreover, the temperature window is very narrow, and the deviation of the temperature by 20 °C leads to structure destruction. The ordered assemblies are obtained only for low coverages, where the formation of quasi-one-dimensional chains is observed [17]. For larger coverage, the molecular assemblies are unordered and even three-dimensional, with only very limited areas exhibiting the herringbone-like pattern. This indicates that the key role is played by the kinetics of the processes, and the equilibration of the system easily destroys the ordered molecular structures [41]. A summary of the observed molecular assemblies for PTCDA molecules on rutile titania surfaces is presented in Table 1.

**Table 1:** Molecular structures formed by PTCDA molecules under different conditions on TiO_2_(110)-(1 × 1) and TiO_2_(011)-(2 × 1).

conditions	TiO_2_(110)	TiO_2_(011)

deposited at room temperature	for coverage up to 0.7 ML formation of meandering molecular chains; for coverage larger than 0.7 ML, creation of a well-ordered brick-wall-like pattern	for low coverage, single molecules; for larger coverage, unordered assemblies
annealing at 100 °C	for coverage up to 0.7 ML, transformation from meandering molecular chains into ordered brick-wall-like islands; for coverage larger than 0.7 ML, improvement of the layer quality	no significant influence
annealing at 150 °C	destruction of the ordered structures	no significant influence
deposition at 100 °C	creation of a well-ordered brick-wall-like pattern	formation of well-ordered molecular chains running along surface rows

Similar results were obtained for other small (i.e., terephthalic acid, TPA) molecules [41], which form well-ordered molecular layers on the TiO_2_(110) surface and unordered structures on the (011) face. Additionally, molecules belonging to a different family of large polycyclic molecules, i.e., phthalocyanines (Pc), exhibit remarkably different behaviour on the two faces. The experiments showed that copper phthalocyanines (CuPc) form ordered layers on the (011) face and completely unordered assemblies on the (110) surface [41]. All of these observations point to significant differences in chemical properties between the two (110) vs (011) faces of rutile titania, which are exhibited through completely different molecule behaviour.

## Experimental

The measurements were performed in an ultra-high vacuum (UHV) system containing preparation, analytical, radial distribution and microscope chambers. In the experiments, a commercially available variable-temperature STM (VT-STM), manufactured by Omicron Nanotechnology GmbH, was used. The base pressure in the UHV system was in the low 10^−10^ mbar range. The preparation of samples was performed by the application of the standard procedure containing sputtering with argon ions and thermal annealing [36]. To obtain well-reconstructed large terraces, the last preparation cycle was followed by slow cooling of the sample. The surface quality was monitored with a low-energy electron diffraction (LEED) setup. PTCDA molecules were evaporated from a standard Knudsen cell at approximately 580 K on the substrate maintained at room or elevated temperature. The molecular flux was controlled using a quartz microbalance. Before the measurements, the sample was cooled to approximately 100 K using a flow cryostat, and the STM imaging was performed in a constant current mode at positive bias voltages (empty state imaging) with etched tungsten tips used as probes. For image processing and STM data analysis, WSxM software was used [42].
